# Ibrahim Pacha and His Doctor's Bill

**Published:** 1846-09

**Authors:** 


*Ibrahim Pacha and his Doctor's Bill.*—It is generally known that Ibrahim Pacha spent a few months in the south of France for the benefit of his health, and that an eminent medical man, Dr. Lallemand, left his practice for a considerable time to attend on the son of Mehemet Ali. Nothing was arranged as to the fees or the salary of the M. D., until two or three days before Ibrahim Pacha was to leave Paris, and then he sent 50,000 francs (2,000*l.*) to Dr. Lallemand. This sum was not considered satisfactory by Dr. Lallemand, who had been building castles with the munificent sum he expected from the Pacha, and he therefore respectfully submitted that he considered his fees should be estimated at 200,000 francs (8,000*l.*) It was a source of great vexation to Ibrahim to have undervalued the services of Dr. Lallemand, yet he did not make up the donation named, but sent 4,000*l.* more to the learned physician, who then declared himself satisfied with the total received, *videlicet*, 6,000*l.*—*London Exam.*

